# Identification and replication of novel genetic variants of ABO gene to reduce the incidence of diseases and promote longevity by modulating lipid homeostasis

**DOI:** 10.18632/aging.203700

**Published:** 2021-11-22

**Authors:** Xiaolin Ni, Chen Bai, Chao Nie, Liping Qi, Yifang Liu, Huiping Yuan, Xiaoquan Zhu, Liang Sun, Qi Zhou, Yan Li, Hefu Zhen, Huabing Su, Rongqiao Li, Rushu Lan, Guofang Pang, Yuan Lv, Wei Zhang, Fan Yang, Yao Yao, Chen Chen, Zhaoping Wang, Danni Gao, Nan Zhang, Shenqi Zhang, Li Zhang, Zhu Wu, Caiyou Hu, Yi Zeng, Ze Yang

**Affiliations:** 1 The Key Laboratory of Geriatrics, Beijing Institute of Geriatrics, Beijing Hospital, National Center of Gerontology, National Health Commission, Institute of Geriatric Medicine, Chinese Academy of Medical Sciences, Beijing 100730, P.R. China; 2 Graduate School of Peking Union Medical College, Chinese Academy of Medical Science, Beijing 100730, P.R. China; 3 Center for the Study of Aging and Human Development and Geriatrics Division, Medical School of Duke University, Durham, NC 27708, USA; 4 Center for Healthy Aging and Development Studies, National School of Development, Peking University, Beijing 100871, P.R. China; 5 BGI-Shenzhen, Shenzhen, Guangdong 518083, P.R. China; 6 College of Science and Technology, Hebei Agricultural University, Cangzhou 061100, Hebei, P.R. China; 7 Joint Graduate Program of Peking-Tsinghua-NIBS, School of Life Sciences, Tsinghua University, Beijing 100084, P.R. China; 8 Jiangbin Hospital, Guangxi Zhuang Autonomous Region, Nanning 530021, P.R. China; 9 Peking University Fifth School of Clinical Medicine, Beijing 100191, P.R. China

**Keywords:** healthy longevity, ABO, plasma lipid levels, O-linked glycosylation

## Abstract

Genes related to human longevity have not been studied so far, and need to be investigated thoroughly. This study aims to explore the relationship among ABO gene variants, lipid levels, and longevity phenotype in individuals (≥90yrs old) without adverse outcomes. A genotype-phenotype study was performed based on 5803 longevity subjects and 7026 younger controls from the Chinese Longitudinal Healthy Longevity Survey (CLHLS). Four ABO gene variants associated with healthy longevity (rs8176719 C, rs687621 G, rs643434 A, and rs505922 C) were identified and replicated in the CLHLS GWAS data analysis and found significantly higher in longevity individuals than controls. The Bonferroni adjusted *p-value* and OR range were 0.013-0.020 and 1.126-1.151, respectively. According to the results of linkage disequilibrium (LD) analysis, the above four variants formed a block on the ABO gene (D’=1, r^2^_range_ = 0.585-0.995). The carriers with genotypes rs687621 GG, rs643434 AX, or rs505922 CX (p_range_ = 2.728 x 10^-107^-5.940 x 10^-14^; OR_range_ = 1.004-4.354) and haplotype CGAC/XGXX (p = 2.557 x 10^-27^; OR = 2.255) had a substantial connection with longevity, according to the results of genetic model analysis. Following the genotype and metabolic phenotype analysis, it has been shown that the longevity individuals with rs687621 GG, rs643434 AX, and rs505922 CX had a positive association with HDL-c, LDL-c, TC, TG (p_range_ = 2.200 x 10^-5^-0.036, OR_range_ = 1.546-1.709), and BMI normal level (p_range_ = 2.690 x 10^-4^-0.026, OR_range_ = 1.530-1.997). Finally, two pathways involving vWF/ADAMTS13 and the inflammatory markers (sE-selectin/ICAM1) that co-regulated lipid levels by glycosylation and effects on each other were speculated. In conclusion, the association between the identified longevity-associated ABO variants and better health lipid profile was elucidated, thus the findings can help in maintaining normal lipid metabolic phenotypes in the longevity population.

## INTRODUCTION

A healthy life span is a complex phenotype that is influenced by both genetic and environmental factors. It has been observed that the influence of genetic factors increases with age [1]. Based on recent genetic studies, more than 50 different genes are associated with longevity in different populations [2–6]. Many reported studies have revealed that individuals with a life span of ≥ 90 years had several healthy genetic variants, indicating the importance of genetic contribution to a longer life span. Some of these variants were found to be associated with plasma lipid homeostasis that could delay the onset or prevent diseases and promote a longer life span [4].

The balance between metabolism and plasma lipids is vital for physiological turnover. The results of the Long Life Family Study (LLFS), an international collaborative study, showed that individuals with a longer life span had a better lipid profile [7, 8]. The molecular composition and concentration of lipid species are indicative of their cellular localization, metabolism, and, consequently, their impact on age-related diseases and a healthy life span [9]. Previous studies have identified a few loci associated with longevity involving lipid metabolisms, such as APOE Ɛ2, TOMM40 rs2075650, FOXO3A rs2802292, CETP rs5882, HLA-DQB1 rs1049107, and rs1049100 in individuals with an exceptionally long life span [10–13].

Recently, our group has successively reported some lipid metabolism-related genetic variances associated with a healthy life span. However, the overall genetic basis of these variances is unidentified, and given this, there may be more yet unexplained genetic variances whose cumulative influence increases longevity by altering and maintaining lipid homeostasis [10–13]. There are multiple gene interaction networks in our body, which together maintain the body's physiological balance, including lipid metabolism. We tried to find more genetic variants that promoted longevity and metabolic balance to explain their biological significance through multi-gene network interaction.

Many studies have shown that the ABO gene has been linked to longevity [14–16]. Fortney et al., (2015) evaluated and replicated five loci including rs514659 in ABO in Caucasians by applying informed genome-wide association studies (iGWAS) [17]. Timmers et al. used a genome-wide association (GWA) of 1 million parental lifespans of genotyped subjects and data on mortality risk factors to identify and replicate rs2519093 in ABO in the English population [18]. But it is still not clear for ABO variants in longevity in other populations, for example, Chinese. So, it is important to develop this study in Chinese to confirm ABO variants associated with human longevity.

In addition, using NGS, other ABO SNPs, which were potential causal loci related to lipid homeostasis and health, were discovered subsequently. Previous research suggested that individuals with the ABO genotype, i.e., rs8176719 CC, had improved overall cardiovascular health and increased longevity via plasma lipid levels [14, 19–21]. According to a meta-analysis of the LURIC and YFS cohorts, the minor allele of Ars657152 of the ABO gene was significantly associated with greater cholesterol absorption that results in disrupted healthy aging [22]. Another research found that the major rs644234*T allele of the ABO gene was associated with decreased levels of apolipoprotein E (ApoE), a multifunctional protein involved in lipid metabolism and longevity [23–25]. Hence, it is needed to identify some loci on the ABO gene associated with longevity and lipid metabolism. So far, there are few reports on genetic variants of the ABO gene and plasma lipids associated with healthy longevity. Meanwhile, the genetic mechanism by which ABO gene variants protect against lipid metabolic disorders and promote healthy aging is unknown.

Hence, the current study explored the ABO gene genetic variants that maintain plasma lipid homeostasis and enhance health longevity. Based on the CLHLS, a population genetic analysis was conducted in the Chinese population to find genetic variants of the ABO gene linked to a long life span and normal plasma lipid levels. We used genome-wide association studies (GWAS), metabolic phenomics technology, and combined analysis to identify the possible beneficial variants by performing a comparative analysis between longevity and age-specific control groups in these population cohorts. The obtained results would offer a new perspective on understanding a healthy longer life span and aging.

## RESULTS

### Identification of new longevity-associated ABO variations

First, the raw data was collected from GWAS phases I and II, and data quality control procedures were followed for the sample screening. There were 5803 longevity subjects and 7026 young controls with genotype left. Then, based on chromosomal position (i.e., chromosome 9: 136125788-136150617) of the ABO gene, 80% of the participants including 4437 longevity individuals and 5627 young controls with genotype were randomly selected to identify variants on ABO genes.

**Figure 1:**
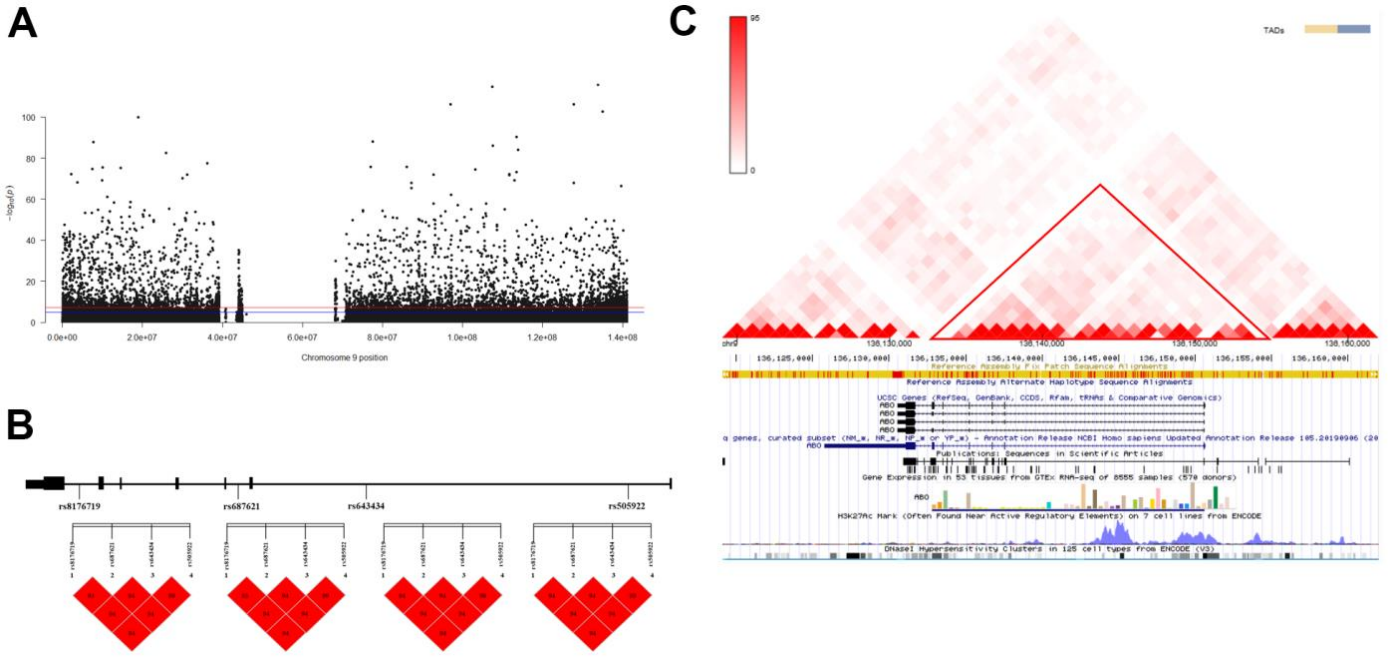
**Association analysis identified ABO as the longevity-associated gene.** (**A**) Manhattan plot of Genome-Wide Association Studies (GWAS) on chromosome 9; (**B**) Linkage Disequilibrium (LD) analysis of the four variants. a: LD map of centenarians; b: LD map of nonagenarians; c: LD map of longevity; d: LD map of young controls. (**C**) Interaction analysis of the four variants in the three-dimensional genome. The red triangle box shows the Topologically Associating Domains (TAD) region on the ABO gene.

**Figure 2:**
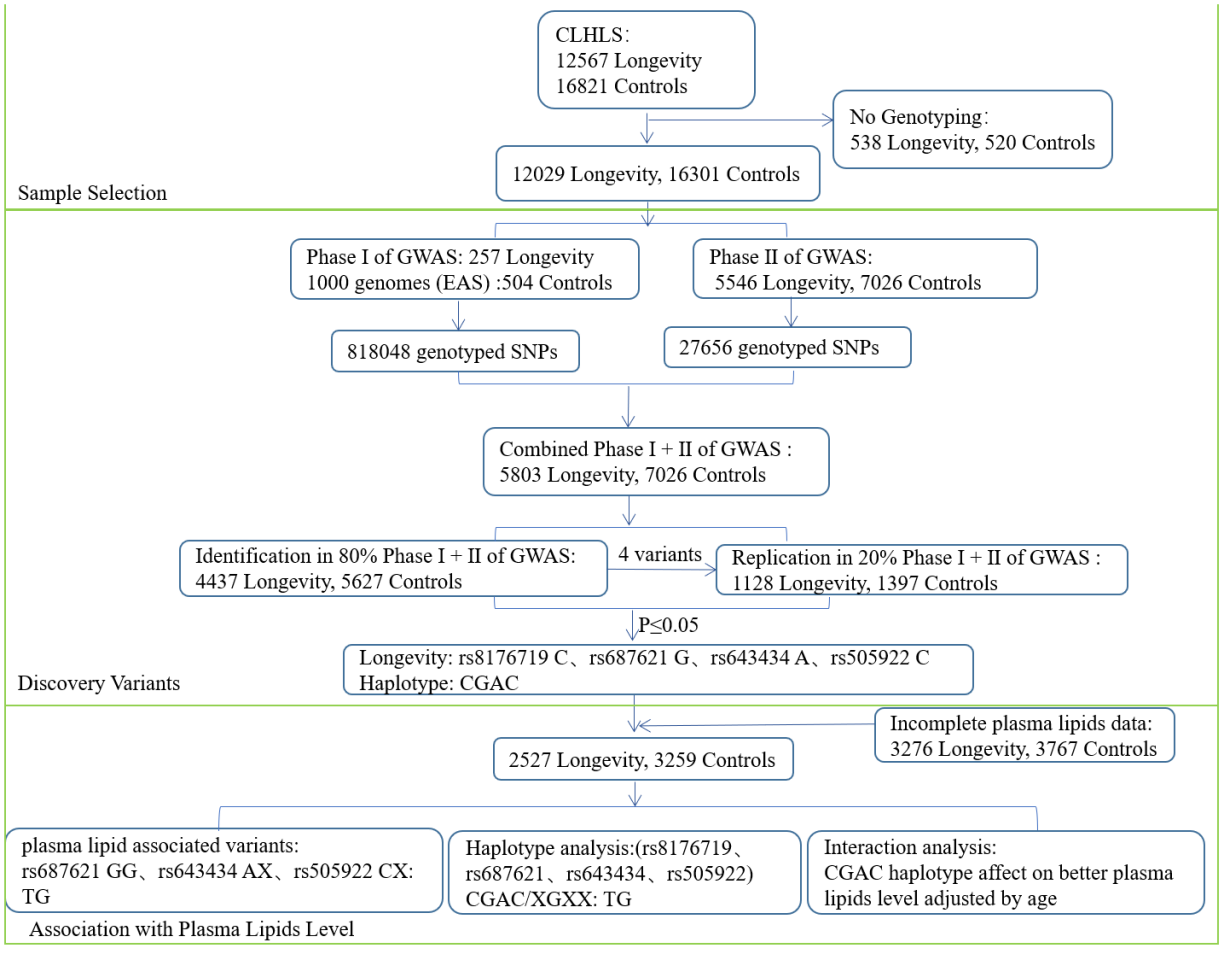
A flow chart of the consecutive analysis steps.

Seven variants (i.e., rs8176722, rs8176719, rs687621, rs2519093, rs514659, rs643434, and rs505922) were genotyped on ABO genes and four of them were associated with longevity (p≤0.05) as shown in Figure 1A. While the flowchart for the steps of sequential analytical has been shown in Figure 2.

### Replication of the longevity-associated variants

Herein, four variants have been verified with 20% of participants involved in 1128 longevity subjects and 1397 young controls with genotype. One reported rs8176719 (p _Bonferroni_ _genotype_ = 0.016, p _Bonferroni_ _allele_ = 0.013, OR _allele_ = 1.151, 95%CI _allele_: 1.018-1.302) [16], and three novel variants in the ABO gene showed significant differences both in the allele and the genotype frequencies while comparing longevity and younger controls. Three variants including rs687621 (p _Bonferroni_ _genotype_ = 0.006, p _Bonferroni_ _allele_ = 0.018, OR _allele_ = 1.131, 95%CI _allele_: 1.008-1.268), rs643434 (p _Bonferroni_ _genotype_ = 0.008, p _Bonferroni_ _allele_ = 0.016, OR _allele_ = 1.131, 95%CI _allele_: 1.010-1.267), and rs505922 (p _Bonferroni_ _genotype_ = 0.002, p _Bonferroni_ _allele_ = 0.020, OR _allele_ = 1.126, 95%CI _allele_: 1.006-1.260) were identified in accordance with Hardy-Weinberg equilibrium in the younger controls (p > 0.05), and were positively correlated with longevity (p ≤ 0.05) (Table 1 and Supplementary Table 1).

**Table 1:** Identification and replication of ABO variants in healthy longevity.

**Gene**	**ID(Ref/Alt)**	**Group**	**Phase I + phase II of GWAS (80%)**								
			**Major homo**	**Hetro**	**Minor homo**	**P _Bonferroni_**	**Major allele**	**Minor allele**	**P _Bonferroni_**	**OR**	**95%IC**
			**Case/control**	**Case/control**	**Case/control**		**Case/control**	**Case/control**			
ABO	rs8176719(-/C)	Longevity/Control	1342/1570	1838/2419	684/894	0.020	4522/5559	3206/4207	0.017	1.067	1.005-1.134
	rs687621(A/G)	Longevity/Control	1423/1759	1904/2648	744/1013	0.018	4750/6166	3392/4674	0.022	1.062	1.002-1.125
	rs643434(G/A)	Longevity/Control	1593/1885	2047/2700	797/1036	0.023	5233/6470	3641/4772	0.022	1.060	1.002-1.122
	rs505922(T/C)	Longevity/Control	1597/1896	2033/2698	794/1033	0.020	5227/6490	3621/4764	0.023	1.060	1.001-1.121
**Gene**	**ID**	**Group**	**Phase I + phase II of GWAS (20%)**								
			**Major homo**	**Hetro**	**Minor homo**	**P _Bonferroni_**	**Major allele**	**Minor allele**	**P _Bonferroni_**	**OR**	**95%IC**
			**Case/control**	**Case/control**	**Case/control**		**Case/control**	**Case/control**			
ABO	rs8176719(-/C)	Longevity/Control	337/417	447/570	140/239	0.016	1121/1404	727/1048	0.013	1.151	1.018-1.302
	rs687621(A/G)	Longevity/Control	360/408	554/608	185/280	0.006	1274/1424	924/1168	0.018	1.131	1.008-1.268
	rs643434(G/A)	Longevity/Control	389/464	561/663	167/270	0.008	1339/1591	895/1203	0.016	1.131	1.010-1.267
	rs505922(T/C)	Longevity/Control	376/452	579/651	173/283	0.002	1331/1555	925/1217	0.020	1.126	1.006-1.260

### Identification of longevity-associated haplotypes

The results of both LD analysis and three-dimensional (3D) genome interaction revealed a block formed by rs8176719, rs687621, rs643434, and rs505922 variants on the ABO gene (D’=1, r^2^_range_ = 0.585-0.995, Figure 1B, 1C). The CGAC haplotype enhanced the probability of longevity (*p-value* = 4.926 x 10^-17^, OR: 1.315, 95% CI: 1.233-1.401), as compared to the-AGT haplotype. Furthermore, as compared to the-AGT haplotype, the CGAC haplotype was correlated with both nonagenarians (*p-value* = 1.589 x 10-3, OR: 1.127, 95% CI: 1.046-1.214) and centenarians (*p-value* = 3.460 x 10^-4^, OR: 1.18, 95% CI: 1.078-1.291) (Table 2).

**Table 2:** Haplotype analysis of rs8176719, rs687621, rs643434 and rs505922.

**Haplotype**	**Longevity**	**Control**	**P**	**OR**	**95%CI**
-AGT	3964	4720			
CGAC	3503	3173	4.926*10^-17^	1.315	1.233-1.401
	Nonagenarians	Control			
-AGT	2558	4720			
CGAC	1938	3173	1.589*10^-3^	1.127	1.046-1.214
	Centenarians	Control			
-AGT	1406	4720			
CGAC	1115	3173	3.460*10^-4^	1.180	1.078-1.291

### Longevity–associated variants were independent of APOE e3, and e2.

APOE e2 is associated with significantly increased odds of longevity [26]. The layered results of APOE alleles indicated that there were four haplotypes with frequencies > 0.03. A comparison between longevity and young controls revealed that the CGAC haplotypes (*p*_e3_=1.340 x 10^-10^, OR_e3_ = 1.285, 95%CI_e3_: 1.190-1.387) and (*p*_e2_ = 2.720 x 10^-4^, OR_e2_ = 1.320, 95%CI_e2_: 1.137-1.533) were associated with longevity in either APOE e3 or e2. Therefore, the CGAC haplotypes increased the likelihood of attaining a longevity age independently (Supplementary Table 3).

### Genotypes and haplotype in genetic model analysis

According to genetic model analysis, the carriers, along with genotypes and phenotype haplotype, i.e., rs687621 GG (*p-value* = 2.728 x 10^-107^, OR = 4.341, 95%CI: 3.775-4.992), rs643434 AX (AG+AA) (*p-value* = 8.271 x 10^-26^, OR = 1.497, 95%CI: 1.388-1.614), rs505922 CX (CT+CC) (*p-value* = 8.354 x 10^-26^, OR = 1.497, 95%CI: 1.388-1.614), and CGAC/XGXX (CGAC/-GGT+ CGAC/CGAC) (*p-value* = 2.557 x 10^-27^, OR = 2.255, 95%CI: 1.940-2.621) were found to be significantly associated with longevity (Table 3). The longer-lived populations were then divided into nonagenarians and centenarians, who have been compared to young controls individually. Three variants, i.e., rs687621 GG, rs643434 AX (AG+AA), and rs505922 CX (CT+CC) (*p*_range_ = 5.940 x 10^-14^ - 2.187 x 10^-95^, OR _range_ = 1.460-4.354), and haplotype genotype CGAC/XGXX (CGAC/-GGT+CGAC/CGAC) (*p*_range_ = 1.458 x 10^-18^ - 3.466 x 10^-22^, OR _range_ = 2.224-2.310) were all associated with nonagenarians and centenarians (Table 3).

**Table 3:** Genotypes and haplotype in genetic model analysis.

**Variants**		**Case/control**	**Case/control**	**P**	**OR**	**95%CI**
rs687621	Genotype	GG	AX			
	Longevity/Control	929/279	4241/5529	2.728*10^-107^	4.341	3.775-4.992
	Nonagenarians/Controls	630/279	2880/5529	2.187*10^-95^	4.335	3.739-5.026
	Centenarians/Controls	299/279	1361/5529	1.441*10^-70^	4.354	3.660-5.179
	Centenarians/Nonagenarians	299/630	1361/2880	0.956	1.004	0.863-1.169
rs643434	Genotype	AX	GG			
	Longevity/Control	3572/3173	1982/2635	8.271*10^-26^	1.497	1.388-1.614
	Nonagenarians/Controls	2428/3173	1378/2635	5.134*10^-19^	1.463	1.345-1.591
	Centenarians/Controls	1144/3173	604/2635	5.940*10^-14^	1.532	1.370-1.713
	Centenarians/Nonagenarians	1144/2428	604/1378	0.452	1.047	0.929-1.179
rs505922	Genotype	CX	TT			
	Longevity/Control	3579/3182	1973/2626	8.354*10^-26^	1.497	1.388-1.614
	Nonagenarians/Controls	2430/3182	1374/2626	9.168*10^-19^	1.460	1.342-1.587
	Centenarians/Controls	1149/3182	599/2626	4.988*10^-16^	1.583	1.416-1.770
	Centenarians/Nonagenarians	1149/2430	599/1374	0.181	1.085	0.963-1.222
Haplotype of rs8176719, rs687621, rs643434, rs505922	Genotype	CGAC/XGXX	-AGT/-XGT			
	Longevity/Control	541/279	4091/4758	2.557*10^-27^	2.255	1.940-2.621
	Nonagenarians/Controls	342/279	2622/4758	3.466*10^-22^	2.224	1.886-2.624
	Centenarians/Controls	199/279	1469/4758	1.458*10^-18^	2.310	1.908-2.797
	Centenarians/Nonagenarians	199/342	1469/2622	0.690	1.039	0.862-1.251

Note: X represents the major allele or minor allele of the corresponding SNP.

### Genotype-phenotype study of longevity-associated variants and plasma lipid or BMI

There were 2,527 longevity subjects with an average age of 96.06 years and 3,259 young controls with an average age of 70.00 years in the samples with integrated epidemiological data. CLHLS participants were 1455 nonagenarians and 1072 centenarians. Sex, disease history, BMI, plasma lipids, blood pressure, and blood glucose were compared between different age groups. We found a statistical difference in the distribution of sex (p = 1.575 x 10^-57^), BMI (p = 2.359 x 10^-3^), and lipid levels (p = 8.000 x 10^-5^) between longevity and young controls (Supplementary Table 4).

In the normal plasma lipid and the BMI group, the recessive model GG of rs687621 (*p*_lipid_ = 0.036, OR _lipid_ = 1.709, 95%CI_lipid_: 1.031-2.834; *p*_BMI_ = 0.026, OR _BMI_ = 1.997, 95%CI _BMI_: 1.077-3.706), the dominant model AX (AG+AA) of rs643434 (*p*_lipid_ = 2.200 x 10^-5^, OR _lipid_ = 1.550, 95%CI _lipid_: 1.264-1.891; *p*_BMI_ = 2.690 x 10^-4^, OR _BMI_ = 1.530, 95%CI _BMI_: 1.216-1.924), and CX (CT+CC) of rs505922 (*p*_lipid_ = 2.200 x 10^-5^, OR _lipid_ = 1.546, 95%CI _lipid_: 1.264-1.891; *p*_BMI_ = 2.690 x 10^-4^, OR _BMI_ = 1.530, 95%CI _BMI_: 1.216-1.924), were positively correlated with plasma lipid and BMI separately. On combining the normal plasma lipid and the BMI levels, the dominant model GG of rs687621 (*p* = 0.038, OR = 2.106, 95%CI: 1.027-4.319), the recessive model AX (AG+AA) of rs643434 (*p* = 7.590 x 10^-3^, OR = 1.450, 95%CI: 1.103-1.905), and CX (CT+CC) of rs505922 (*p* = 7.590 x 10^-3^, OR = 1.450, 95%CI: 1.103-1.905) also showed significant differences compared with the young controls (Table 4).

**Table 4:** Plasma lipids and BMI analysis in different genotype group.

**SNP**	**rs687621**				
**Genotype**	**GG**	**AG+AA**	**P**	**OR**	**95%CI**
Longevity/Control					
Lipids (-)					
Longevity	34	644	0.036	1.709	1.031-2.834
Control	29	939			
BMI (-)					
Longevity	23	480	0.026	1.997	1.077-3.706
Control	19	792			
Lipids (-)+BMI (-)					
Longevity	19	354	0.038	2.106	1.027-4.319
Control	13	510			
**SNP**	**rs643434**				
**Genotype**	**GA+AA**	**GG**	**P**	**OR**	**95%CI**
Longevity/Control					
Lipids (-)					
Longevity	436	242	2.200*10^-5^	1.550	1.264-1.891
Control	521	447			
BMI (-)					
Longevity	326	177	2.690*10^-4^	1.530	1.216-1.924
Control	443	368			
Lipids (-)+BMI (-)					
Longevity	240	133	7.590*10^-3^	1.450	1.103-1.905
Control	290	233			
**SNP**	**rs505922**				
**Genotype**	**TC+CC**	**TT**	**P**	**OR**	**95%CI**
Longevity/Control					
Lipids (-)					
Longevity	436	242	2.200*10^-5^	1.546	1.264-1.891
Control	521	447			
BMI (-)					
Longevity	326	177	2.690*10^-4^	1.530	1.216-1.924
Control	443	368			
Lipids (-)+BMI (-)					
Longevity	240	133	7.590*10^-3^	1.450	1.103-1.905
Control	290	233			

Note: (-) represents the normal level of plasma lipids or BMI.

### Relationship between longevity-associated variants and plasma lipid homeostasis

The analysis of the lipid metabolism index (HDL-c, LDL-c, TG, and TC) showed that the longevity samples possessed lower LDL-c levels (*p-value* = 1.700 x 10^-5^), TG (*p-value* = 1.275 x 10^-22^), and TC (*p-value* = 0.011). There were significant differences in the levels of LDL-c (*p-value* = 7.669 x 10^-7^), TG (*p-value* = 2.522 x 10^-16^), and TC (*p-value* = 6.400 x 10^-5^) between nonagenarians and the young controls. Only two indices, TG (*p-value* = 2.941 x 10^-13^) and HDL (*p-value* = 0.049) showed significant differences between centenarians and the young controls. TG was a common difference index in comparison between the different age groups (Supplementary Table 5).

Next, we analyzed the subgroups of plasma lipid levels in both longevity samples and the young controls. Based on the criteria for plasma lipid levels, the rs687621 AG genotype (*p-value* = 0.018, OR = 1.638, 95%CI: 1.085-2.473), the rs643434 GA genotype (*p-value* = 0.016, OR = 1.651, 95%CI: 1.096-2.488), and the rs505922 TC genotype (*p* = 0.016, OR = 1.651, 95%CI: 1.096-2.488) were significantly increased with normal TG levels in the longevity subjects. The rs687621 G allele carriers showed better TG levels compared with the A allele carriers (*p-value* = 0.042, OR = 1.387, 95%CI: 1.012-1.901) (Supplementary Table 8).

**Figure 3:**
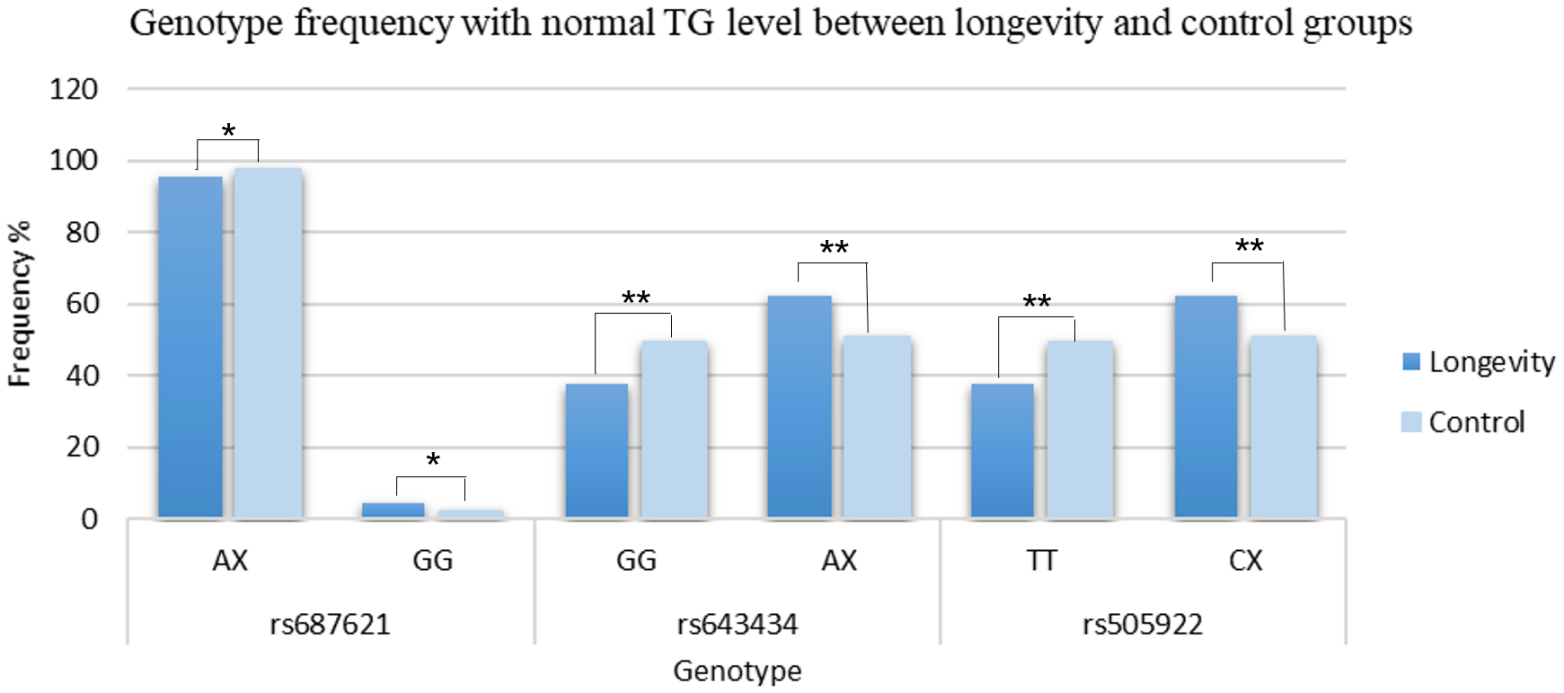
**Comparison of genotype frequencies between the longevity and the control group.** *p≤0.05; **p≤0.01.

The recessive model GG of rs687621 (*p* = 0.044, OR = 1.620, 95%CI: 1.008-2.604), the dominant model AX (AG+AA) of rs643434 (*p* = 3.977 x 10^-7^, OR = 1.612, 95%CI: 1.340-1.940), and CX (CT+CC) of rs505922 (*p* = 3.977 x 10^-7^, OR = 1.612, 95%CI: 1.340-1.940) were positively correlated with normal TG levels consistently (Figure 3 and Supplementary Table 9).

## DISCUSSION

### Identification of longevity-associated variants and haplotypes

Longevity is a highly complicated phenotype that is influenced by genetic as well as environmental factors. The various cut-off to define longevity have been used, varying from 85+, 90+ and 100+ years, and the impact of these differences have been addressed in Broer’s paper (2015) [3]. In this study, the longevity phenotype is considered as individuals (≥90yrs old) without major health complications, including CVD, cancer, diabetes, hypertension, etc. Individuals that have a longer life span with a lower risk of aging-associated diseases are regarded as a model of healthy aging. Our previous genetic research has identified some longevity-associated factors, such as FOXO3 [27], IGFBP-3 [28], CETP [29], SIRT1 [30], and HLA-DQB1 [10].

According to the reported studies, ABO has been associated with blood transfusions, organ transplants, and diseases such as cancer, coronary heart disease (CHD), and lower circulating cholesterol levels [31–34]. However, after multiple GWAS database analyses, Fortney et al. proposed the ABO may be associated with longevity [17]. We hypothesized that there are some ABO variations associated with longevity in Chinese.

In our cohort, we identified and replicated four SNPs in the ABO gene that were associated with healthy aging and longevity, including rs8176719, rs687621, rs643434, and rs505922, and three of these variants have never been identified in previous studies on longevity. Compared with the young controls, all four variants showed a significant difference in longevity, which suggested that these four variants were longevity-associated genetic variances that could increase the lifespan by healthy aging. Next, by analyzing 5803 longevity subjects and 7026 young controls, we showed that a single-nucleotide insertion in codon 87 (rs8176719) constructed a strong linkage disequilibrium block (LD; r^2^ = 0.944) between rs687621, rs643434, and rs505922 in the ABO gene. This is the first study to report that rs8176719 C, rs687621 G, rs643434 A, rs505922 C (*p*_Bonferroni_ _range_ = 0.013-0.020; OR _range_ = 1.126-1.151) and the CGAC (p = 4.926 x 10^-17^, OR = 1.315) significantly increased the probability of healthy life with a longer life span (Tables 1, 2). The results of genetic model analysis showed that individuals carrying rs687621 GG, rs643434 AX (AG+AA), rs505922 CX (CT+CC) (*p*_range_ = 2.728 x 10^-107^-5.940 x 10^-14^; OR _range_ = 1.004-4.354), and CGAC/XGXX (CGAC/-GGT+ CGAC/CGAC) (*p* = 2.557 x 10^-27^; OR = 2.255) were also significantly associated with longevity.

Our study focused on ABO variants associated with longevity in Chinese. We have identified three novel variants (rs687621, rs643434, and rs505922) of the ABO gene different from Caucasians and replicated one allele (rs8176719) in ABO reported before [17, 18]. The obtained results revealed that ABO gene variants are associated with human longevity, but there existed many different variants in the ABO gene among different populations.

### Longevity variants associated with lipid homeostasis in individuals with a longer life span

Many longevity-associated variants were found that were potentially associated with maintaining the balance of plasma lipids. Several observational studies have found that increases in TG levels are associated with an increase in the risk of morbidity and mortality related to aging-associated diseases [35, 36]. In the Leiden Longevity Study (LLS), lower levels of TG, one of the biomarkers of healthy aging, were found to decrease morbidity associated with aging-related disorders [37, 38].

In this study, we found that these novel longevity-associated variants were also healthy-lipid-associated variants, as the longevity individuals with ABO rs687621, rs643434, and rs505922 were significantly associated with normal lipid (*p*_range_ = 2.200 x 10^-5^-0.036, OR _range_ = 1.546-1.709) and normal BMI level (*p*_range_ = 2.690 x 10^-4^-0.026, OR _range_ = 1.530-1.997) (Table 4 and Supplementary Figure 1).

Hence, we identified ABO variants associated with two phenotypes in the Chinese population: longevity and normal lipid levels. Considering the potential bias existed in the selection of longevity and local control individuals for analysis, we compared the major demographic and characteristics of the participants between the included (2527 longevity, 3259 controls) and excluded (3276 longevity, 3767 controls). Meanwhile, we also compared them of the participants between the included (2527 longevity, 3259 controls) and total (5803 longevity, 7026 controls). There was not statistically significance between any pair’s comparison identified (Supplementary Table 6). Therefore, we justify our included subjects (2527 longevity, 3259 controls) are equally balanced or objectively represented with all participants of ours. Besides, we did stratification analysis of lipid metabolism by genotype and age, and also showed that there was no selection bias (Supplementary Tables 7–10). Therefore, we hypothesized that there was a significant correlation between ABO variants and longevity and lipid normal levels in the Chinese population, which needs further investigation.

### Functional analysis of the new healthy-associated variants in ABO

The ABO gene (chromosome 9q34.2) is known to determine the presence of antigens on the surface of red blood cells. Our results showed that except for rs8176719, the other three novel SNPs, i.e., rs687621, rs643434, and rs505922 that were identified in our study were all located in the intron region. Data from ENCODE showed that rs687621 was located in a region featured by enhancer histone marks and could act as an expression Quantitative Trait Locus (eQTL). It was possible that the expression of ABO was being increased by other variants proxied by rs687621 [39]. The other two SNPs, i.e., rs643434 and rs505922, located in intron 1 of the ABO gene were highly linked (LD; r^2^ = 0.994). Noncoding transcript exon variant rs8176719 was a frameshift mutation in exon 6. Because of a potential open chromatin region, several epigenetic markers, a transcription factor binding site, and evolutionary conservation, the combined prediction results from ENCODE, ChIP-seq, and UCSC suggested that rs8176719 might be crucial for gene regulation [40].

The glycosylation of soluble cell adhesion molecules links the ABO blood group antigens to E-selectin ligand-1 and P-selectin glycoprotein ligand-1 [41]. ABO SNPs altered lipid levels by working on the clearance and glycosylation of membrane molecules, including biomarkers (such as soluble cell adhesion molecules: sE-selectin, sP-selectin, ICAM1) [42]. Glycosylation can occur on the ligand itself, the receptor, as well as on key signaling enzymes and effector proteins. Regarding the glycosylation of lipids, the process of O-linked glycosylation, which is generally initiated by the addition of the monosaccharide, i.e., N-acetylgalactosamine to the hydroxyl group of serine and threonine amino acids (GalNAca1-O-Ser/Thr) is critical for the LDL receptor stability, and stable expression of the very low-density lipoprotein receptors on the cell surface. Interaction analysis of genes revealed an interaction relationship between ABO and ADAMTS13, as represented in Figure 1 (Supplementary Figure 2). Some studies indicated that individuals carrying rs8176719 CC have plasma levels of von Willebrand Factor (VWF) 25% lower than individuals carrying rs8176746 A allele due to increased proteolysis and clearance of VWF at the Tyr1605-Met1606 bond by ADAMTS13 [20, 43], which specifically inhibits platelet deposition and inflammation, and reducing the risk of death [41].

**Figure 4:**
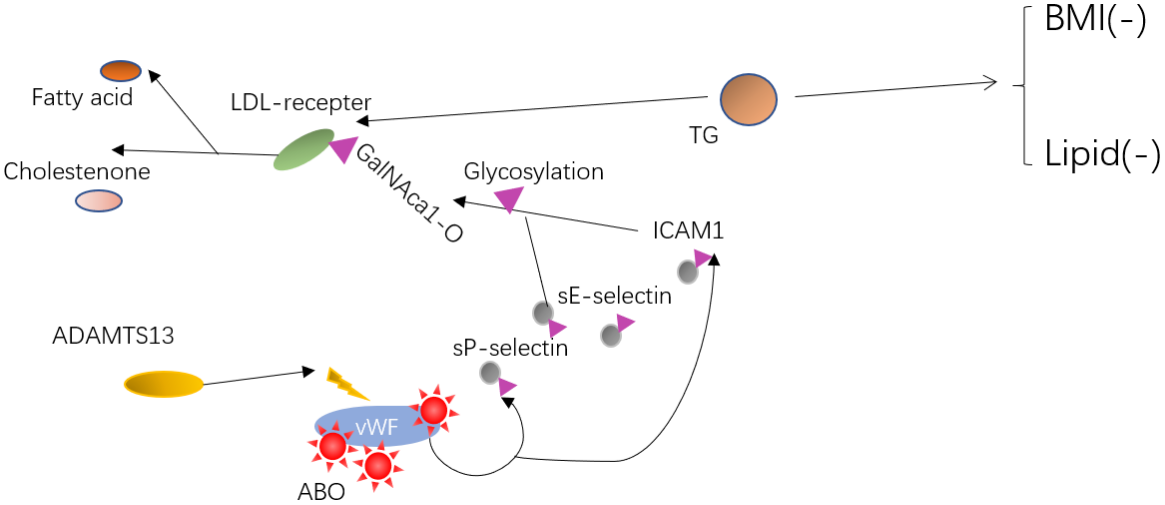
**The possible mechanism or interactive pathway from relevant information on ABO and plasma lipids phenotype.** Mechanism of action for ABO variants may result in the vWF/ADAMTS13 and sE-selectin/ICAM1 functional change. Lastly, two pathways involving vWF/ADAMTS13 and the inflammatory markers (sE-selectin/ICAM1) that co-regulated lipid levels by O-linked glycosylation and effects on each other were speculated.

Individuals carrying rs687621, rs643434, and rs505922 altered TG concentrations by glycosylating the target molecules using the O-linked sugar domain, and may stabilize circulating inflammatory markers and lipid levels by promoting healthy lipid metabolism, thus contributing to individual healthy longevity (Figure 4).

Hence, we identified and replicated the presence of four longevity-associated variants in our cohort, as well as a new haplotype (on the ABO gene) linked to longevity. Then, the analysis of genotype and metabolic phenotypes showed that the longevity individuals with rs687621 GG, rs643434 AX (AG+AA), and rs505922 CX (CT+CC) were associated with normal levels of lipid and BMI. Lastly, two pathways involving vWF/ADAMTS13 and the inflammatory markers that co-regulated lipid levels by glycosylation and effects on each other were speculated. As a result, we can deduce that individuals with longevity-associated variants have an improved cardiovascular profile, which may lower the risk of aging-related disorders and maintain healthy physical circumstances, resulting in longer life. Although we indicated the relationship between the ABO blood group and healthy longevity, several pieces of evidence involved the mechanism of ABO blood group antigens and lipids metabolism. Additionally, healthy longevity could be studied in cell or animal models by using new technologies, such as single-cell sequencing, CRISPR/Cas 9, and 3D organ models. Thus, we need to understand the mechanism of longevity and achieve healthy aging for all human communities.

## CONCLUSIONS

The present study revealed that rs8176719 C, rs687621 G, rs643434 A, and rs505922 C of the ABO gene were not only longevity-associated genetic variants but also lipid homeostasis-associated variants in our cohort. These variants probably altered triglyceride concentrations by glycosylation on the target molecules by the O-linked sugar domain, and promoted healthy lipid metabolism, thereby contributing to longevity. Our results showed that ABO longevity-associated genotypes (rs687621 GG, rs643434 AX, and rs505922 CX) could promote lipid homeostasis. In the future, further functional and mechanism studies should be conducted to better understand the molecular mechanism of longevity associated with ABO and lipid homeostasis.

## MATERIALS AND METHODS

### Subjects

All experimental procedures were reviewed and approved by the Ethics Committee of Beijing Hospital, Ministry of Health, China. We obtained written consent forms from all participants before study initiation. All clinical investigations were conducted following the principles of the Declarations of Helsinki.

The Chinese Longitudinal Healthy Longevity Surveys (CLHLS), which enrolled in 1998, 2000, 2002, 2005, 2008, 2011, and 2014 in a randomly selected half of the counties and cities in 22 out of 31 provinces in China, provided the samples for this study, which included 12567 people with a longer life span and 16821 young controls. The CLHLS covers approximately 85% of the total population of China. We interviewed all consented longevity in the sampled counties and cities. Young middle-aged controls (30–85 years old) were obtained in the same country/city as long-lived individuals, who needed to satisfy one specific criterion of having a non-family history of longevity (no lineal family members within three generations aged above 85) [4, 44].

### DNA extraction and genotyping

DNA was extracted from the whole blood and hybridized following the manufacturer’s instructions. A total of 257 longevity individuals (aged 102.04±2.05 years) were genotyped using the Illumina HumanOmniZhongHua-8 Bead Chips, that were created by strategically selecting optimized tag SNP content from all three HapMap phases and the 1000 Genomes Project (1 kGP). The chip represents a state-of-the-art choice for GWAS in Asian populations to maximize international compatibility [4]. After standard GWAS quality-control filtering for subjects, we obtained a total of 818048 genotyped SNPs.

The phase II of GWAS was from 5546 longevity subjects (97.66±4.96 years) and 7026 young controls (aged 67.72±13.65 years) in CLHLS. Based on previous research, phase II of GWAS used a custom SNP chip with 27,656 selected longevity and disease-related SNPs for targeted genotyping [44].

### Genome-wide association analysis

We combined the raw data from GWAS phases I and II and completed the sample filtering data quality control procedures. There were 5803 longevity subjects and 7026 young controls with genotype. Then we randomly selected 80% and 20% of participants with genotype for discovery and validation, respectively.

All data from GWAS were analyzed by PLINK (v1.06) [4]. Genotypic distributions of all single nucleotide polymorphisms (SNPs) in the population were analyzed based on the Hardy-Weinberg Equilibrium (HWE) (all *p*-values > 0.05) (Supplementary Table 2).

Laboratory parameters and genotypic data of the present GWAS were from CLHLS, which were offered by the Center for Healthy Aging and Development Studies, National School of Development, Peking University.

### Selection of variations and genotyping

We identified the variants associated with longevity from 80% of GWAS phase I + II samples (4437 longevity subjects and 5627 young controls) by the chromosomal location of the ABO gene (chromosome 9: 136125788-136150617). There were 4 variants in the ABO gene identified as candidate variants with a MAF (minor allele frequency) greater than 10%. We included duplicate samples with 1128 longevity subjects and 1397 young controls as quality controls to verify the reliability of the variants (Supplementary Table 11). Finally, four genetic variants were identified as longevity-associated genetic variants. The multiple comparisons of CLHLS GWAS phase I and II study of our group were performed the Bonferroni correction. P-value thresholds ≤ 0.025 were considered significant. The Haploview software was used to perform haplotype analysis. The 3D Genome Browser (http://3dgenome.fsm.northwestern.edu/) was used to examine three-dimensional genome interactions.

### Association of variation and metabolic genotype in longevity

In CLHLS, there were 2527 individuals (aged 90-114 years) and 3259 young controls (aged 38-85 years). Both sets included an integrated questionnaire of an epidemiological survey as well as biochemical indexes. (Supplementary Table 4). Laboratory parameters, including blood pressure, high-density lipoprotein-cholesterol (HDL-c), low-density lipoprotein-cholesterol (LDL-c), total cholesterol (TC), triglyceride (TG), body mass index (BMI), and blood glucose (FBG) were recorded. The normal plasma lipids and BMI levels are according to the guide and reported at home and abroad (FPG _normal_=2.80-5.60 mmol/L; BMI _normal_=18.5-25; TC _normal_≤5.18 mmol/L; TG _normal_≤1.70 mmol/L; HDL _normal_≥1.04 mmol/L; LDL _normal_≤3.37 mmol/L) [45–48]. The relationships of alleles, genotypes, and haplotypes with phenotypes were studied individually using univariate or multifactorial stratification analysis, as applicable.

### Genetic model analysis

Long-lived individuals carry special mutations associated with longevity. The base sequence of the gene has been changed (partially or completely) in longevity compared to a normal individual. A variation in the degrees of association between the genotypes and phenotype of the risk and non-risk SNPs has been clearly understood. Therefore, according to Mendel's mode of inheritance, we compared the frequency of longevity and controls who carries mutations or not. The strength of association between the genotypes and phenotype was estimated using the odds ratio (OR). P-value threshold≤0.05 was considered statistically significant and p≤0.01 was considered extremely significant.

### Statistical analysis

The Statistical Package for Social Sciences (SPSS Inc, Chicago, IL, USA) Windows, v 19.0 was used for statistical analysis. Gene counting was done to determine the differences in the distribution of genotype and allele frequencies, and the χ2 goodness-fit test was used to test the deviations from the Hardy-Weinberg equilibrium (HWE) for all SNPs. The odds ratio (OR) was used to estimate the strength of association between the variables, with 95% confidence intervals (95%CI). *p*≤0.05 was considered statistically significant. The mean and standard deviation (SD) were used to describe the normally distributed plasma lipid levels as continuous variables.

### Availability of data and material

The datasets generated during and/or analyzed during the current study are available from the corresponding author on reasonable request.

## Supplementary Material

Supplementary Figures

Supplementary Tables 1-7

Supplementary Table 8

Supplementary Tables 9-11
