# Supplementary material for: Identification and replication of novel genetic variants of ABO gene to reduce the incidence of diseases and promote longevity by modulating lipid homeostasis
Source: Aging (Albany NY). 2021 Nov 22;13(22):24655–74. doi: 10.18632/aging.203700 (PMC8660604; doi:10.18632/aging.203700)
Supplement: Supplementary Figures [file aging-13-203700-s001.pdf]

## SUPPLEMENTARY FIGURES

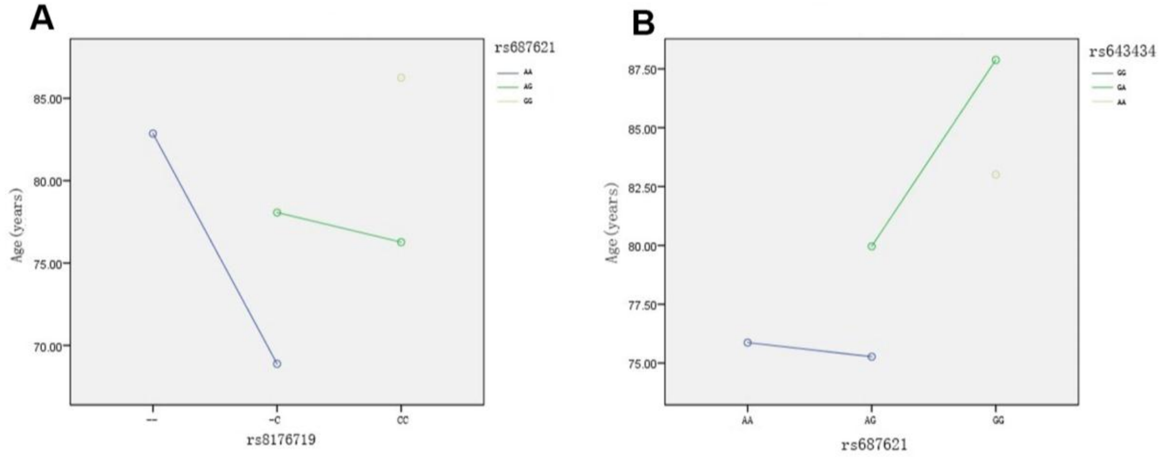


**Supplementary Figure 1. Interaction analysis between plasma lipids level and variants on age.** ABO longevity variant allele carriers take a trend of better blood lipid homeostasis (**A**) interaction analysis between rs8176719 and rs687621 (**B**) interaction analysis between rs687621, rs643434 and TG.

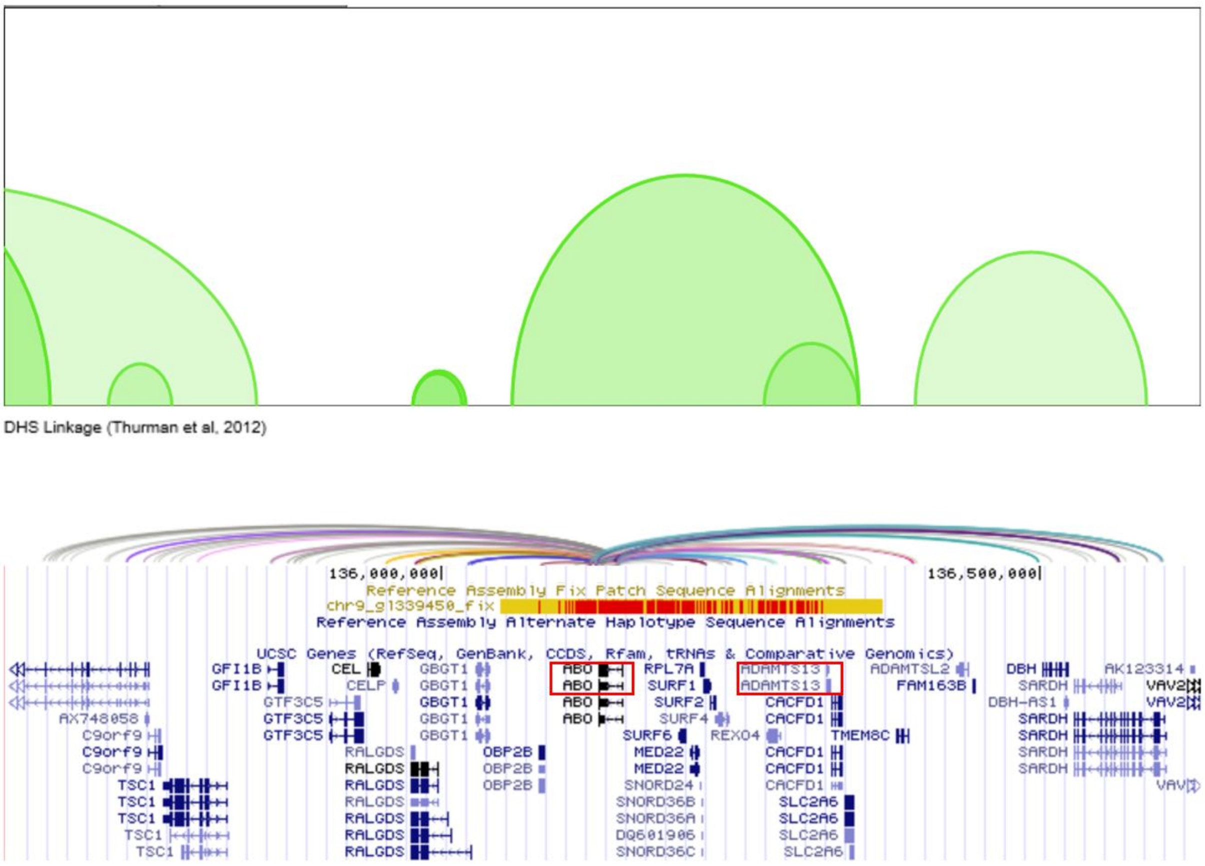


**Supplementary Figure 2. Interaction analysis between ABO and ADAMTS13.** The green arc shows the interaction between ABO and ADAMTS13 genes.
