# Supplementary material for: Identification and replication of novel genetic variants of ABO gene to reduce the incidence of diseases and promote longevity by modulating lipid homeostasis
Source: Aging (Albany NY). 2021 Nov 22;13(22):24655–74. doi: 10.18632/aging.203700 (PMC8660604; doi:10.18632/aging.203700)
Supplement: Supplementary Tables 1-7 [file aging-13-203700-s002.pdf]

SUPPLEMENTARY TABLES

Supplementary Table 1. Replication of ABO variants in healthy longevity.

Gene	ID(Ref/Alt)	Group	Phase I + phase II of GWAS								
			Major homo Case/control	Hetro Case/control	Minor homo Case/control	P Bonferroni	Minor allele Case/control	Major allele Case/control	P Bonferroni	OR	95%IC
ABO	rs8176719(-/C)	Longevity/Control	1679/1987	2285/2989	824/1133	0.007	3933/5255	5643/6963	0.002	1.083	1.026-1.143
		Nonagenarians/Controls	1098/1987	1460/2989	534/1133	0.007	2528/5255	3656/6963	0.003	1.091	1.026-1.161
		Centenarians/Controls	581/1987	825/2989	290/1133	0.126	1405/5255	1987/6963	0.049	1.067	0.988-1.153
		Centenarians/Nonagenarians	581/1098	825/1460	290/534	0.307	1405/2528	1987/3656	0.304	0.978	0.898-1.065
	rs687621(A/G)	Longevity/Control	1783/2167	2458/3256	929/1293	0.012	4316/5842	6024/7590	0.004	1.074	1.020-1.131
		Nonagenarians/Controls	1229/2167	1651/3256	630/1293	0.008	2911/5842	4109/7590	0.003	1.086	1.025-1.152
		Centenarians/Controls	554/2167	807/3256	299/1293	0.227	1405/5842	1915/7590	0.111	1.049	0.971-1.133
		Centenarians/Nonagenarians	554/1229	807/1651	299/630	0.240	1405/2911	1915/4109	0.206	0.966	0.888-1.050
	rs643434(G/A)	Longevity/Control	1982/2349	2608/3363	964/1306	0.011	4536/5975	6572/8061	0.003	1.074	1.021-1.130
		Nonagenarians/Controls	1378/2349	1765/3363	663/1306	0.007	3091/5975	4521/8061	0.003	1.084	1.024-1.147
		Centenarians/Controls	604/2349	843/3363	301/1306	0.185	1445/5975	2051/8061	0.083	1.052	0.976-1.134
		Centenarians/Nonagenarians	604/1378	843/1765	301/663	0.202	1445/3091	2051/4521	0.235	0.97	0.895-1.053
	rs505922(T/C)	Longevity/Control	1973/2348	2612/3356	967/1316	0.013	4546/5988	6558/8052	0.003	1.073	1.020-1.128
		Nonagenarians/Controls	1374/2348	1765/3356	665/1316	0.008	3095/5988	4513/8052	0.003	1.084	1.025-1.148
		Centenarians/Controls	599/2348	847/3356	302/1316	0.180	1451/5988	2045/8052	0.110	1.048	0.972-1.130
		Centenarians/Nonagenarians	599/1374	847/1765	302/665	0.162	1451/3095	2045/4513	0.206	0.967	0.891-1.048

Supplementary Table 2. Hardy-Weinberg equilibrium of ABO variants in cases and controls.

P	rs8176719	rs687621	rs643434	rs505922
Longevity	0.329	0.106	0.036	0.043
Control	0.879	0.263	0.094	0.057

Supplementary Table 3. Haplotype analysis of rs8176719, rs687621, rs643434 and rs505922 by APOE allele.

APOE	Haplotype	Longevity	Control	P	OR	95%CI
e3e3	-AGT	3297.00(0.59)	3940.00(0.57)	0.047	1.075	1.001-1.155
	CAGT	172.98(0.03)	689.00(0.10)	$2.361*10^{-46}$	0.300	0.252-0.357
	CGGT	254.02(0.05)	326.01(0.05)	0.617	0.958	0.810-1.133
	CGAC	1870.98(0.33)	1937.99(0.28)	$1.340*10^{-10}$	1.285	1.190-1.387
e2	-AGT	914.00(0.59)	1002.00(0.56)	0.105	1.121	0.976-1.287
	CAGT	52.00(0.03)	204.00(0.12)	$2.340*10^{-18}$	0.269	0.196-0.367
	CGGT	74.00(0.05)	93.00(0.05)	0.560	0.911	0.666-1.246
	CGAC	503.00(0.33)	475.99(0.27)	$2.720*10^{-4}$	1.320	1.137-1.533
e4	-AGT	582.00(0.60)	812.00(0.56)	0.073	1.163	0.986-1.372
	CAGT	31.00(0.03)	153.00(0.11)	$1.770*10^{-11}$	0.278	0.187-0.413
	CGGT	35.00(0.04)	58.00(0.04)	0.604	0.893	0.582-1.370
	CGAC	321.00(0.33)	417.00(0.29)	0.030	1.215	1.019-1.449

Supplementary Table 4. Base information of population in longevity and controls.

Characteristic	Groups				Longevity vs. control			Nonagenarians vs. control			Centenarians vs. control			Nonagenarians vs. centenarians		
	Controls	Longevity	Nonagenarians	Centenarians	P-value	OR	95% CI	P-value	OR	95% CI	P-value	OR	95% CI	P-value	OR	95% CI
N[a]	3259	2527	1455	1072												
Mean Age(yr)	70.000± 11.854	96.059± 5.470	93.000± 3.238	102.000± 2.235												
Sex																
Male	1067	369	288	105	$1.575*10^{-57}$	3.359	2.887-3.907	$5.539*10^{-25}$	2.425	2.046-2.876	$8.048*10^{-65}$	6.324	5.028-7.955	$6.063*10^{-14}$	2.608	2.021-3.364
Female	805	935	527	501												
Disease History																
No	1738	1100	679	528	0.128	0.744	0.507-1.091	0.283	0.783	0.500-1.226	0.119	0.658	0.388-1.118	0.584	0.841	0.452-1.566
Yes	85	40	26	17												
BMI																
Normal	997	749	504	319	$2.359*10^{-3}$	0.736	0.604-0.897	$1.960*10^{-4}$	0.641	0.507-0.811	0.497	0.917	0.713-1.179	0.019	1.430	1.061-1.928
Abnormal	358	198	116	105												
Lipids																
Normal	1193	926	576	436	$8.000*10^{-5}$	0.703	0.602-0.821	$3.950*10^{-4}$	0.721	0.602-0.864	$8.100*10^{-4}$	0.662	0.539-0.813	0.481	0.918	0.723-1.165
Abnormal	649	354	226	157												
Blood pressure																
Normal	844	546	354	255	0.088	1.133	0.981-1.307	0.473	1.063	0.900-1.255	0.222	1.123	0.932-1.352	0.614	1.057	0.853-1.308
Abnormal	1014	743	452	344												
Blood glucose																
Normal	1685	1166	721	553	0.827	1.027	0.806-1.310	0.322	1.148	0.873-1.510	0.223	0.810	0.577-1.137	0.068	0.706	0.485-1.027
Abnormal	173	123	85	46												

a: N, number.

Supplementary Table 5. Base line of plasma lipids in different age groups.

	N (centenarians)	Mean± Std	N (nonagenarians)	Mean± std	N (controls)	Mean± std	N (longevity)	Mean± std	P (longevity vs. controls)	P (centenarians vs. controls)	P (nonagenarians vs. controls)	P (centenarians vs. nonagenarians)
HDL	581	1.271± 0.311	979	1.245± 0.345	2228	1.239± 0.365	1560	1.255± 0.333	0.154	0.049	0.595	0.172
LDL	581	2.407± 0.859	979	2.300± 0.833	2228	2.459± 0.834	1560	2.340± 0.844	$1.700*10^{-5}$	0.182	$7.669*10^{-7}$	0.015
TG	581	1.034± 0.598	979	1.061± 0.720	2228	1.384± 1.211	1560	1.051± 0.677	$1.275*10^{-22}$	$2.941*10^{-13}$	$2.522*10^{-16}$	0.625
TC	581	4.163± 1.058	979	3.947± 1.145	2228	4.126± 1.197	1560	4.027± 1.118	0.011	0.492	$6.400*10^{-5}$	$4.040*10^{-4}$

N, number.

Supplementary Table 6. The comparison of phenotype selection bias.

Group		$N_{Phenotype\ (included)}$		$N_{Phenotype\ (excluded)}$		N_{Total}		Phenotype (included) vs. Total		Phenotype (included) vs. phenotype (excluded)	
		Longevity	Control	Longevity	Control	Longevity	Control	$P_{longevity}$	$P_{control}$	$P_{longevity}$	$P_{control}$
Sex	Male	115	885	254	182	369	1067	0.588	0.727	0.460	0.239
	Female	272	684	663	121	935	805				
rs8176749	--	142	498	1537	1489	1679	1987	0.784	0.298	0.751	0.130
	-C	182	800	2103	2189	2285	2989				
	CC	63	271	761	862	824	1133				
	-	466	1796	5177	5167	5643	6963	0.487	0.805	0.451	0.749
	C	308	1342	3625	3913	3933	5255				
rs687621	AA	168	595	1615	1572	1783	2167	0.594	0.387	0.536	0.197
	AG	210	884	2248	2372	2458	3256				
	GG	78	321	851	972	929	1293				
	A	546	2074	5478	5516	6024	7590	0.345	0.235	0.302	0.118
	G	366	1526	3950	4316	4316	5842				
rs643434	GG	194	638	1788	1711	1982	2349	0.687	0.348	0.636	0.165
	GA	235	913	2373	2450	2608	3363				
	AA	87	321	877	985	964	1306				
	G	623	2189	5949	5872	6572	8061	0.452	0.254	0.409	0.134
	A	409	1555	4127	4420	4536	5975				
rs505922	TT	193	638	1780	1710	1973	2348	0.717	0.289	0.669	0.120
	TC	236	912	2376	2444	2612	3356				
	CC	88	321	879	995	967	1316				
	T	622	2188	5936	5864	6558	8052	0.493	0.218	0.452	0.105
	C	412	1554	4134	4434	4546	5988				
Lipids	Normal	678	968	248	225	926	1193	0.756	0.568	0.422	0.088
	Abnormal	267	505	87	144	354	649				
BMI	Normal	503	811	246	186	749	997	0.299	0.934	0.328	0.798
	Abnormal	151	289	86	69	198	358				

N, number.

Supplementary Table 7. Association between genotype and plasma lipid levels in longevity and controls.

	Longevity				Control			
**rs8176719**	**HDL**	**LDL**	**TG**	**TC**	**HDL**	**LDL**	**TG**	**TC**
--	1.270±0.361	2.320±0.879	1.131±0.789	4.091±1.195	1.244±0.365	2.465±0.825	1.478±1.189	4.041±1.252
-C	1.274±0.334	2.432±0.935	1.131±0.746	4.138±1.247	1.257±0.358	2.545±0.865	1.468±1.420	4.161±1.297
CC	1.292±0.340	2.439±0.935	1.097±0.849	4.184±1.231	1.209±0.375	2.456±0.822	1.412±1.331	4.191±1.187
P(-- VS.CC)	0.430	0.664	0.652	0.387	0.237	0.271	0.350	0.721
P(-C VS.CC)	0.387	0.446	0.571	0.466	0.201	0.376	0.709	0.784
-C+CC	1.279±0.336	2.434±0.934	1.122±0.774	4.150±1.242	1.245±0.363	2.523±0.855	1.454±1.397	4.169±1.269
P(-C+CC VS.--)	0.123	0.817	0.674	0.457	0.914	0.524	0.321	0.682
rs687621								
AA	1.217±0.324	2.228±0.825	1.201±0.918	3.850±1.196	1.235±0.356	2.448±0.827	1.598±1.278	3.916±1.371
AG	1.267±0.321	2.414±0.900	1.059±0.634	4.143±1.127	1.256±0.367	2.553±0.849	1.360±1.338	4.266±1.174
GG	1.204±0.281	2.176±0.820	0.925±0.436	3.856±1.006	1.152±0.379	2.267±0.951	1.709±1.766	4.157±1.188
P(AA VS.GG)	0.867	0.484	0.961	0.768	0.009	0.271	0.16	0.435
P(AG VS.GG)	0.514	0.457	0.644	0.406	0.007	0.006	0.086	0.108
AG+GG	1.263±0.319	2.397±0.897	1.049±0.623	4.124±1.120	1.251±0.368	2.539±0.856	1.376±1.362	4.261±1.175
P(AG+GG VS.AA)	0.122	0.847	0.394	0.449	0.826	0.442	0.097	0.511
rs643434								
GG	1.217±0.324	2.224±0.827	1.198±0.918	3.845±1.198	1.240±0.360	2.488±0.862	1.605±1.528	3.998±1.379
GA	1.264±0.322	2.400±0.903	1.053±0.631	4.126±1.133	1.254±0.367	2.525±0.827	1.326±1.104	4.258±1.134
AA	1.235±0.247	2.409±0.695	0.994±0.378	4.162±0.688	1.054±0.347	2.251±0.975	1.329±1.165	3.907±1.083
P(GG VS.AA)	0.966	0.690	0.823	0.804	0.013	0.289	0.358	0.988
P(GA VS.AA)	0.832	0.850	0.338	0.974	$3.000*10^{-6}$	$9.890*10^{-4}$	0.873	0.041
GA+AA	1.263±0.319	2.400±0.895	1.051±0.623	4.127±1.118	1.250±0.367	2.519±0.831	1.326±1.105	4.251±1.134
P(GA+AA VS.GG)	0.133	0.860	0.377	0.440	0.686	0.498	0.056	0.421
rs505922								
TT	1.217±0.324	2.224±0.827	1.198±0.918	3.845±1.198	1.240±0.360	2.488±0.862	1.605±1.528	3.998±1.379
TC	1.264±0.322	2.400±0.903	1.053±0.631	4.126±1.133	1.254±0.367	2.526±0.827	1.327±1.104	4.260±1.134
CC	1.235±0.247	2.409±0.695	0.994±0.378	4.162±0.688	1.067±0.340	2.194±0.973	1.271±1.513	3.837±1.088
P(TT VS.CC)	0.966	0.690	0.823	0.804	0.034	0.060	0.477	0.896
P(TC VS.CC)	0.832	0.850	0.338	0.974	$2.200*10^{-5}$	0.004	0.867	0.045
TC+CC	1.263±0.319	2.400±0.895	1.051±0.623	4.127±1.118	1.250±0.367	2.519±0.831	1.326±1.105	4.251±1.134
P(TC+CC VS.TT)	0.133	0.860	0.377	0.440	0.686	0.498	0.056	0.421
