# Supplementary material for: Identification and replication of novel genetic variants of ABO gene to reduce the incidence of diseases and promote longevity by modulating lipid homeostasis
Source: Aging (Albany NY). 2021 Nov 22;13(22):24655–74. doi: 10.18632/aging.203700 (PMC8660604; doi:10.18632/aging.203700)
Supplement: Supplementary Table 8 [file aging-13-203700-s003.docx]

**Supplementary Table 8. Association between genotype and plasma lipids level in different group.**

| SNP |  | Longevity | | | | Controls | | | |
| --- | --- | --- | --- | --- | --- | --- | --- | --- | --- |
|  |  | HDL | | P | OR(95%CI) | HDL | | P | OR(95%CI) |
|  |  | ≥1.04 | <1.04 |  |  | ≥1.04 | <1.04 |  |  |
| rs8176719 | N(--) | 274 | 91 | ref |  | 351 | 143 | ref |  |
|  | N(-C) | 364 | 110 | 0.562 | 1.099(0.799-1.512) | 559 | 226 | 0.952 | 1.008(0.786-1.292) |
|  | N(CC) | 130 | 40 | 0.725 | 1.079(0.705-1.653) | 181 | 88 | 0.279 | 0.838(0.608-1.154) |
|  | N(CC+-C) | 494 | 150 | 0.557 | 1.094(0.811-1.476) | 740 | 314 | 0.734 | 0.960(0.759-1.214) |
|  | N(--+-C) | 638 | 201 | 0.717 | 1.054(0.793-1.402) | 910 | 369 | 0.968 | 1.005(0.799-1.263) |
|  | N(-) | 912 | 292 | ref |  | 1261 | 512 | ref |  |
|  | N(C) | 624 | 190 | 0.638 | 1.052(0.853-1.296) | 921 | 402 | 0.363 | 0.930(0.796-1.087) |
| rs687621 | N(AA) | 237 | 91 | ref |  | 421 | 176 | ref |  |
|  | N(AG) | 415 | 129 | 0.184 | 1.235(0.904-1.688) | 643 | 263 | 0.850 | 1.022(0.815-1.283) |
|  | N(GG) | 30 | 10 | 0.714 | 1.152(0.541-2.452) | 27 | 18 | 0.138 | 0.627(0.337-1.168) |
|  | N(GG+AG) | 445 | 139 | 0.188 | 1.229(0.904-1.672) | 670 | 281 | 0.978 | 0.997(0.796-1.248) |
|  | N(AA+AG) | 652 | 220 | 0.376 | 1.138(0.855-1.515) | 1064 | 439 | 0.901 | 1.013(0.823-1.248) |
|  | N(A) | 889 | 311 | ref |  | 1485 | 615 | ref |  |
|  | N(G) | 475 | 149 | 0.342 | 1.115(0.891-1.396) | 697 | 299 | 0.676 | 0.965(0.819-1.138) |
| rs643434 | N(GG) | 238 | 91 | ref |  | 530 | 222 | ref |  |
|  | N(GA) | 424 | 136 | 0.265 | 1.192(0.875-1.624) | 553 | 227 | 0.857 | 1.020(0.819-1.272) |
|  | N(AA) | 20 | 3 | 0.126 | 2.549(0.740-8.785) | 8 | 8 | 0.077 | 0.419(0.155-1.130) |
|  | N(AA+GA) | 444 | 139 | 0.202 | 1.221(0.898-1.661) | 561 | 235 | 1 | 1.000(0.804-1.244) |
|  | N(GG+GA) | 662 | 227 | 0.453 | 1.115(0.839-1.482) | 1083 | 449 | 0.916 | 1.010(0.834-1.224) |
|  | N(G) | 900 | 318 | ref |  | 1613 | 671 | ref |  |
|  | N(A) | 464 | 142 | 0.215 | 1.155(0.920-1.449) | 569 | 243 | 0.769 | 0.974(0.818-1.161) |
| rs505922 | N(TT) | 238 | 91 | ref |  | 530 | 222 | ref |  |
|  | N(TC) | 424 | 136 | 0.265 | 1.192(0.875-1.624) | 552 | 227 | 0.870 | 1.019(0.817-1.269) |
|  | N(CC) | 20 | 3 | 0.126 | 2.549(0.740-8.785) | 9 | 8 | 0.118 | 0.471(0.179-1.237) |
|  | N(CC+TC) | 444 | 139 | 0.202 | 1.221(0.898-1.661) | 561 | 235 | 1.000 | 1.000(0.804-1.244) |
|  | N(TT+TC) | 662 | 227 | 0.453 | 1.115(0.839-1.482) | 1082 | 449 | 0.924 | 1.009(0.834-1.222) |
|  | N(T) | 900 | 318 | ref |  | 1612 | 671 | ref |  |
|  | N(C) | 464 | 142 | 0.215 | 1.155(0.920-1.449) | 570 | 243 | 0.789 | 0.976(0.820-1.163) |
|  |  | LDL | | P | OR(95%CI) | LDL | | P | OR(95%CI) |
|  |  | ≤3.37 | >3.37 |  |  | ≤3.37 | >3.37 |  |  |
| rs8176719 | N(--) | 321 | 44 | ref |  | 425 | 69 | ref |  |
|  | N(-C) | 405 | 69 | 0.293 | 0.805(0.536-1.207) | 667 | 118 | 0.600 | 0.918(0.666-1.265) |
|  | N(CC) | 143 | 27 | 0.224 | 0.726(0.432-1.219) | 234 | 35 | 0.713 | 1.085(0.701-1.680) |
|  | N(CC+-C) | 548 | 96 | 0.208 | 0.782(0.534-1.147) | 901 | 153 | 0.774 | 0.956(0.704-1.299) |
|  | N(--+-C) | 726 | 113 | 0.503 | 0.881(0.607-1.278) | 1092 | 187 | 0.726 | 0.948(0.704-1.277) |
|  | N(-) | 1047 | 157 | ref |  | 1517 | 256 | ref |  |
|  | N(C) | 691 | 123 | 0.187 | 0.842(0.653-1.087) | 1135 | 188 | 0.857 | 1.019(0.831-1.249) |
| rs687621 | N(AA) | 297 | 31 | ref |  | 518 | 79 | ref |  |
|  | N(AG) | 463 | 81 | 0.020 | 0.597(0.385-0.925) | 770 | 136 | 0.335 | 0.863(0.640-1.164) |
|  | N(GG) | 37 | 3 | 0.687 | 1.287(0.375-4.419) | 38 | 7 | 0.659 | 0.828(0.357-1.918) |
|  | N(GG+AG) | 500 | 84 | 0.031 | 0.621(0.402-0.961) | 808 | 143 | 0.324 | 0.862(0.641-1.159) |
|  | N(AA+AG) | 760 | 112 | 0.106 | 0.708(0.465-1.078) | 1288 | 215 | 0.523 | 0.914(0.692-1.206) |
|  | N(A) | 1057 | 143 | ref |  | 1806 | 294 | ref |  |
|  | N(G) | 537 | 87 | 0.216 | 0.835(0.627-1.112) | 846 | 150 | 0.432 | 0.918(0.742-1.136) |
| rs643434 | N(GG) | 298 | 31 | ref |  | 645 | 107 | ref |  |
|  | N(GA) | 478 | 82 | 0.024 | 0.606(0.391-0.940) | 668 | 112 | 0.942 | 0.989(0.743-1.317) |
|  | N(AA) | 21 | 2 | 0.908 | 1.092(0.244-4.880) | 13 | 3 | 0.609 | 0.719(0.201-2.565) |
|  | N(AA+GA) | 499 | 84 | 0.029 | 0.618(0.400-0.956) | 681 | 115 | 0.902 | 0.982(0.739-1.306) |
|  | N(GG+GA) | 776 | 113 | 0.115 | 0.714(0.470-1.086) | 1313 | 219 | 0.966 | 0.995(0.775-1.277) |
|  | N(G) | 1074 | 144 | ref |  | 1958 | 326 | ref |  |
|  | N(A) | 520 | 86 | 0.151 | 0.811(0.609-1.080) | 694 | 118 | 0.857 | 0.979(0.780-1.230) |
| rs505922 | N(TT) | 298 | 31 | ref |  | 645 | 107 | ref |  |
|  | N(TC) | 478 | 82 | 0.024 | 0.606(0.391-0.940) | 667 | 112 | 0.934 | 0.988(0.742-1.315) |
|  | N(CC) | 21 | 2 | 0.908 | 1.092(0.244-4.880) | 14 | 3 | 0.691 | 0.774(0.219-2.739) |
|  | N(CC+TC) | 499 | 84 | 0.029 | 0.618(0.400-0.956) | 681 | 115 | 0.902 | 0.982(0.739-1.306) |
|  | N(TT+TC) | 776 | 113 | 0.115 | 0.714(0.470-1.086) | 1312 | 219 | 0.961 | 0.994(0.774-1.276) |
|  | N(T) | 1074 | 144 | ref |  | 1957 | 326 | ref |  |
|  | N(C) | 520 | 86 | 0.151 | 0.811(0.609-1.080) | 695 | 118 | 0.870 | 0.981(0.781-1.232) |
|  |  | TG | | P | OR(95%CI) | TG | | P | OR(95%CI) |
|  |  | ≤1.7 | >1.7 |  |  | ≤1.7 | >1.7 |  |  |
| rs8176719 | N(--) | 317 | 47 | ref |  | 366 | 128 | ref |  |
|  | N(-C) | 415 | 59 | 0.841 | 1.043(0.692-1.572) | 596 | 189 | 0.459 | 1.103(0.851-1.429) |
|  | N(CC) | 151 | 19 | 0.570 | 1.178(0.668-2.077) | 214 | 55 | 0.091 | 1.361(0.951-1.947) |
|  | N(CC+-C) | 566 | 78 | 0.711 | 1.076(0.731-1.585) | 810 | 244 | 0.236 | 1.161(0.907-1.486) |
|  | N(--+-C) | 732 | 106 | 0.900 | 1.024(0.709-1.479) | 962 | 317 | 0.624 | 1.061(0.837-1.346) |
|  | N(-) | 1049 | 153 | ref |  | 1328 | 445 | ref |  |
|  | N(C) | 717 | 97 | 0.587 | 1.078(0.822-1.414) | 1024 | 299 | 0.107 | 1.148(0.970-1.357) |
| rs687621 | N(AA) | 277 | 50 | ref |  | 424 | 173 | ref |  |
|  | N(AG) | 490 | 54 | 0.018 | 1.638(1.085-2.473) | 718 | 188 | 2.590*10^-4^ | 1.558(1.227-1.979) |
|  | N(GG) | 37 | 3 | 0.186 | 2.226(0.661-7.499) | 34 | 11 | 0.517 | 1.261(0.625-2.546) |
|  | N(GG+AG) | 527 | 57 | 0.013 | 1.669(1.111-2.506) | 752 | 199 | 3.070*10^-4^ | 1.542(1.218-1.952) |
|  | N(AA+AG) | 767 | 104 | 0.123 | 1.331(0.925-1.916) | 1142 | 361 | 0.019 | 1.291(1.043-1.597) |
|  | N(A) | 1044 | 154 | ref |  | 1566 | 534 | ref |  |
|  | N(G) | 564 | 60 | 0.042 | 1.387(1.012-1.901) | 786 | 210 | 8.225*10^-3^ | 1.276(1.065-1.530) |
| rs643434 | N(GG) | 278 | 50 | ref |  | 541 | 211 | ref |  |
|  | N(GA) | 505 | 55 | 0.016 | 1.651(1.096-2.488) | 622 | 158 | 3.570*10^-4^ | 1.535(1.212-1.944) |
|  | N(AA) | 21 | 2 | 0.393 | 1.888(0.429-8.307) | 13 | 3 | 0.411 | 1.690(0.477-5.991) |
|  | N(AA+GA) | 526 | 57 | 0.014 | 1.660(1.105-2.492) | 635 | 161 | 3.130*10^-4^ | 1.538(1.216-1.946) |
|  | N(GG+GA) | 783 | 105 | 0.113 | 1.341(0.932-1.929) | 1163 | 369 | 0.040 | 1.229(1.009-1.498) |
|  | N(G) | 1061 | 155 | ref |  | 1704 | 580 | ref |  |
|  | N(A) | 547 | 59 | 0.060 | 1.354(0.986-1.860) | 648 | 164 | 0.003 | 1.345(1.106-1.635) |
| rs505922 | N(TT) | 278 | 50 | ref |  | 541 | 211 | ref |  |
|  | N(TC) | 505 | 55 | 0.016 | 1.651(1.096-2.488) | 621 | 158 | 3.760*10^-4^ | 1.533(1.210-1.941) |
|  | N(CC) | 21 | 2 | 0.393 | 1.888(0.429-8.307) | 14 | 3 | 0.344 | 1.820(0.518-6.398) |
|  | N(CC+TC) | 526 | 57 | 0.014 | 1.660(1.105-2.492) | 635 | 161 | 3.130*10^-4^ | 1.538(1.216-1.946) |
|  | N(TT+TC) | 783 | 105 | 0.113 | 1.341(0.932-1.929) | 1162 | 369 | 0.041 | 1.228(1.008-1.496) |
|  | N(T) | 1061 | 155 | ref |  | 1703 | 580 | ref |  |
|  | N(C) | 547 | 59 | 0.060 | 1.354(0.986-1.860) | 649 | 164 | 0.003 | 1.348(1.108-1.639) |
|  |  | TC | | P | OR(95%CI) | TC | | P | OR(95%CI) |
|  |  | ≤5.18 | >5.18 |  |  | ≤5.18 | >5.18 |  |  |
| rs8176719 | N(--) | 300 | 64 | ref |  | 409 | 85 | ref |  |
|  | N(-C) | 385 | 89 | 0.657 | 0.923(0.647-1.316) | 637 | 148 | 0.457 | 0.894(0.666-1.200) |
|  | N(CC) | 136 | 34 | 0.501 | 0.853(0.537-1.355) | 226 | 43 | 0.666 | 1.092(0.731-1.631) |
|  | N(CC+-C) | 521 | 123 | 0.552 | 0.904(0.647-1.262) | 863 | 191 | 0.661 | 0.939(0.709-1.244) |
|  | N(--+-C) | 685 | 153 | 0.780 | 0.955(0.692-1.318) | 1046 | 233 | 0.619 | 0.933(0.710-1.226) |
|  | N(-) | 985 | 217 | ref |  | 1455 | 318 | ref |  |
|  | N(C) | 657 | 157 | 0.484 | 0.922(0.734-1.158) | 1089 | 234 | 0.858 | 1.017(0.844-1.225) |
| rs687621 | N(AA) | 286 | 41 | ref |  | 498 | 99 | ref |  |
|  | N(AG) | 446 | 98 | 0.033 | 0.652(0.440-0.967) | 736 | 170 | 0.280 | 0.861(0.655-1.130) |
|  | N(GG) | 37 | 3 | 0.354 | 1.768(0.521-5.996) | 38 | 7 | 0.858 | 1.079(0.468-2.486) |
|  | N(GG+AG) | 483 | 101 | 0.058 | 0.686(0.464-1.014) | 774 | 177 | 0.310 | 0.869(0.663-1.139) |
|  | N(AA+AG) | 732 | 139 | 0.140 | 0.755(0.519-1.098) | 1234 | 269 | 0.475 | 0.912(0.708-1.174) |
|  | N(A) | 1018 | 180 | ref |  | 1732 | 368 | ref |  |
|  | N(G) | 520 | 104 | 0.359 | 0.884(0.679-1.151) | 812 | 184 | 0.519 | 0.938(0.771-1.140) |
| rs643434 | N(GG) | 287 | 41 | ref |  | 617 | 135 | ref |  |
|  | N(GA) | 461 | 99 | 0.041 | 0.665(0.449-0.985) | 641 | 139 | 0.946 | 1.009(0.777-1.310) |
|  | N(AA) | 21 | 2 | 0.591 | 1.500(0.339-6.635) | 14 | 2 | 0.573 | 1.532(0.344-6.818) |
|  | N(AA+GA) | 482 | 101 | 0.054 | 0.682(0.461-1.008) | 655 | 141 | 0.902 | 1.016(0.783-1.319) |
|  | N(GG+GA) | 748 | 140 | 0.156 | 0.763(0.525-1.109) | 1258 | 274 | 0.969 | 1.005(0.800-1.261) |
|  | N(G) | 1035 | 181 | ref |  | 1875 | 409 | ref |  |
|  | N(A) | 503 | 103 | 0.242 | 0.854(0.656-1.112) | 669 | 143 | 0.850 | 1.020(0.827-1.259) |
| rs505922 | N(TT) | 287 | 41 | ref |  | 617 | 135 | ref |  |
|  | N(TC) | 461 | 99 | 0.041 | 0.665(0.449-0.985) | 640 | 139 | 0.956 | 1.007(0.776-1.308) |
|  | N(CC) | 21 | 2 | 0.591 | 1.500(0.339-6.635) | 15 | 2 | 0.510 | 1.641(0.371-7.260) |
|  | N(CC+TC) | 482 | 101 | 0.054 | 0.682(0.461-1.008) | 655 | 141 | 0.902 | 1.016(0.783-1.319) |
|  | N(TT+TC) | 748 | 140 | 0.156 | 0.763(0.525-1.109) | 1257 | 274 | 0.974 | 1.004(0.800-1.260) |
|  | N(T) | 1035 | 181 | ref |  | 1874 | 409 | ref |  |
|  | N(C) | 503 | 103 | 0.242 | 0.854(0.656-1.112) | 670 | 143 | 0.835 | 1.023(0.829-1.261) |

N. number.
