# Supplementary material for: Identification and replication of novel genetic variants of ABO gene to reduce the incidence of diseases and promote longevity by modulating lipid homeostasis
Source: Aging (Albany NY). 2021 Nov 22;13(22):24655–74. doi: 10.18632/aging.203700 (PMC8660604; doi:10.18632/aging.203700)
Supplement: Supplementary Tables 9-11 [file aging-13-203700-s004.pdf]

Supplementary Table 9. Comparison of genotype frequencies with normal TG level between the longevity and the control group.

SNP	Genetic model		Longevity	Control	P	OR	95% CI
			TG≤1.7	TG≤1.7			
rs8176719	Dominant Model	N(--)	317	366	ref		
		N(CC+-C)	566	810	0.023	0.807	0.671-0.971
rs687621	Recessive Model	N(AA+AG)	767	1142	ref		
		N(GG)	37	34	0.044	1.620	1.008-2.604
rs643434	Dominant Model	N(GG)	278	541	ref		
		N(AA+GA)	526	635	$3.977*10^{-7}$	1.612	1.340-1.940
rs505922	Dominant Model	N(TT)	278	541	ref		
		N(CC+TC)	526	635	$3.977*10^{-7}$	1.612	1.340-1.940

N, number.

Supplementary Table 10. Analysis of plasma lipid levels and healthy-associated haplotypes.

	Longevity					Nonagenarians					Centenarians				
**HDL**	**Case**	**Control**	**P**	**OR**	**95%CI**	**Case**	**Control**	**P**	**OR**	**95%CI**	**Case**	**Control**	**P**	**OR**	**95%CI**
-AGT	280	797	-	-	-	147	322	-	-	-	133	475	-	-	-
CAGT	31	92	0.849	0.959	0.624-1.473	18	39	0.971	1.011	0.560-1.827	13	53	0.683	0.876	0.464-1.655
CGAC	142	464	0.244	0.871	0.691-1.099	70	172	0.507	0.891	0.635-1.252	72	292	0.438	0.881	0.638-1.215
LDL															
-AGT	120	957	-	-	-	39	430	-	-	-	81	527	-	-	-
CAGT	23	100	0.014	1.834	1.122-2.998	10	47	0.024	2.346	1.100-5.002	13	53	0.156	1.596	0.833-3.057
CGAC	86	520	0.067	1.319	0.980-1.775	27	215	0.216	1.385	0.825-2.323	59	305	0.215	1.259	0.875-1.811
TG															
-AGT	132	943	-	-	-	67	402	-	-	-	65	541	-	-	-
CAGT	22	101	0.078	1.556	0.948-2.555	15	42	0.018	2.143	1.126-4.079	7	59	0.976	0.987	0.433-2.253
CGAC	59	547	0.115	0.771	0.557-1.066	23	219	0.069	0.630	0.382-1.040	35	328	0.591	0.888	0.576-1.370
TC															
-AGT	157	918	-	-	-	60	409	-	-	-	97	509	-	-	-
CAGT	23	100	0.229	1.345	0.829-2.182	9	48	0.527	1.278	0.597-2.738	14	52	0.279	1.413	0.753-2.649
CGAC	103	503	0.193	1.197	0.913-1.570	37	205	0.358	1.230	0.790-1.915	66	298	0.391	1.162	0.824-1.639
HDL															
CAGT	31	92	-	-	-	18	39	-	-	-	13	53	-	-	-
CGAC	142	464	0.674	0.908	0.580-1.422	70	172	0.693	0.882	0.473-1.645	72	292	0.988	1.005	0.520-1.943
LDL															
CAGT	23	100	-	-	-	10	47	-	-	-	13	53	-	-	-
CGAC	86	520	0.201	0.719	0.433-1.194	27	215	0.188	0.590	0.268-1.302	59	305	0.485	0.789	0.405-1.538
TG															
CAGT	22	101	-	-	-	15	42	-	-	-	7	59	-	-	-
CGAC	59	547	0.009	0.495	0.290-0.844	23	219	$6.000*10^{-4}$	0.294	0.142-0.610	35	328	0.808	0.899	0.382-2.120
TC															
CAGT	23	100	-	-	-	9	48	-	-	-	14	52	-	-	-
CGAC	103	503	0.649	0.890	0.540-1.468	37	205	0.925	0.963	0.435-2.128	66	298	0.554	0.823	0.430-1.572

Supplementary Table 11. Primers of sequencing genotyping.

Variants	Forward primer	Reverse primer	Product length
rs8176719	TGAACTGCTCGTTGAGGATG	GTGGTCAGAGGAGGCAGAAG	185bp
rs687621	GCCACGCACTTCGACCTAT	GGGCTTAGGACCCCGTAAC	782bp
rs643434	CACATTACCTTAGCACCCTT	CTGAGGTGAGAGGATGACTT	432bp
rs505922	AACTGTGTTTGCCATCAAGAAAT	CCCACCATGAAGTGCTTCTC	456bp
